# The bowel loss was preserved with surgical intervention based on multi-item scoring in infants with necrotizing enterocolitis

**DOI:** 10.3389/fsurg.2026.1903124

**Published:** 2026-09-24

**Authors:** Tao Wen, Changsheng Pu, Long Chen, Bijian Liu, Chunbao Guo

**Affiliations:** 1 Department of Pediatric Surgery, Qijiang Health Center for Maternal and Child Care, Chongqing, China; 2 Department of Pediatric Surgery, Chongqing Health Center for Children and Women, Chongqing Medical University, Chongqing, China; 3 Department of Ultrasound, Children's Hospital of Chongqing Medical University, Chongqing, China; 4 Department of Pediatrics, Hechuan Maternal and Child Health Hospital, Chongqing, China

**Keywords:** bowel loss, indicator score, necrotizing enterocolitis, neonate, surgical intervention

## Abstract

**Background:**

Necrotizing enterocolitis (NEC) is a life-threatening gastrointestinal disorder in neonates, and the optimal timing of surgical intervention for this condition remains controversial. This study aimed to determine the appropriate timing of surgery and evaluate its impact on bowel preservation in infants with NEC.

**Methods:**

A multicenter retrospective study was conducted across four hospitals in Chongqing between April 2008 and January 2023. A total of 392 infants diagnosed with NEC (modified Bell stage 2 or higher) were enrolled. All participants were divided into three groups: medical management group (*n* = 192), surgical group with a 13-point indicator score ≤ 5 (*n* = 62), and surgical group with a score > 5 (*n* = 138). The primary outcomes included postoperative intestinal stricture and total bowel resection volume.

**Results:**

Infants in the score > 5 group presented with significantly lower birth weight, gestational age, and body weight at diagnosis (all *P* < 0.01). Compared with the score ≤ 5 group, the score > 5 group had a higher rate of bowel resection (76.1% vs. 58.1%, *P* < 0.001), a higher incidence of ileocecal valve resection (57.2% vs. 19.4%, *P* < 0.001), and a longer median length of resected bowel (19 cm vs. 8 cm, *P* = 0.002). The rates of intestinal stricture (42.0% vs. 17.7%) and total bowel resection (52.2% vs. 21.0%) were also markedly elevated in the score > 5 group. Additionally, this group demonstrated higher rates of 90-day parenteral nutrition dependence, prolonged neonatal intensive care unit (NICU) stay, hospital readmission, and all-cause mortality (all *P* < 0.05).

**Conclusions:**

Surgical intervention based on multi-item scoring was independently associated with reduced bowel resection length in infants with NEC, after adjusting for baseline disease severity and neonatal baseline characteristics.

## Introduction

Necrotizing enterocolitis (NEC) is the most common and severe gastrointestinal emergency predominantly affecting preterm neonates (1, 2). While initial conservative management is the standard approach for most mild-to-moderate NEC cases, 20% to 60% of affected infants require surgical intervention due to progressive clinical deterioration (3, 4).

Clinical strategies for NEC management vary substantially across institutions and individual surgeons, ranging from conservative supportive care to urgent surgical exploration. Multiple attempts have been made to establish standardized criteria for clinical decision-making; nevertheless, the optimal timing of surgery remains a subject of ongoing debate (5, 6). Reported surgical rates for NEC range from 16% to 63% globally, reflecting widespread disagreement among pediatric surgeons regarding the balance between conservative and operative management.

In recent years, bowel loss caused by advanced NEC has attracted increasing clinical attention, as subsequent short bowel syndrome (SBS) leads to long-term nutritional complications and adverse neurodevelopmental outcomes in affected infants (7, 8). Identifying candidates who would benefit from timely surgery to minimize bowel resection is therefore clinically meaningful, yet relevant evidence remains limited. Further investigation into the association between surgical timing and intestinal outcomes is urgently needed to guide individualized management for preterm infants with NEC, and a multidisciplinary treatment model should be implemented based on comprehensive patient assessment.

At our affiliated institutions, NEC management was previously determined by individual surgeon experience. In 2018, we adopted an aggressive surgical protocol for NEC based on published clinical evidence. This retrospective study was designed to explore whether the application of standardized early surgical criteria could reduce bowel loss in infants with NEC.

## Materials and methods

### Study population

This multicenter retrospective study was conducted from April 2008 to January 2023 at four medical institutions in Chongqing: Children's Hospital of Chongqing Medical University, Sanxia Hospital, Yongchuan Maternal and Child Health Care Center, and Jinan Maternal and Child Health Care Hospital. The study protocol was approved by the Institutional Review Board of Chongqing Medical University (Approval No.: CQHCWC-22-168) on April 16, 2022, and the approval covered all participating centers. Given the retrospective nature of the study, the requirement for written informed consent was waived. All clinical data were stored in password-protected encrypted institutional databases, with access restricted exclusively to the research team.

Inclusion criteria: Infants with a confirmed diagnosis of NEC classified as modified Bell stage 2 or higher, based on combined clinical manifestations and radiographic findings. For ambiguous cases, the principal investigator at each center made the final inclusion decision after comprehensive evaluation.

Exclusion criteria: (1) Infants with spontaneous intestinal perforation unrelated to typical NEC; (2) Infants with congenital gastrointestinal malformations, including intestinal atresia, imperforate anus and Hirschsprung disease; (3) Patients who failed to follow the standardized treatment protocol; (4) Cases with incomplete clinical data; (5) Infants who died within 30 days postoperatively due to non-intestinal complications.

### Clinical protocol and surgical indications

Prior to 2017, no unified surgical criteria were available for NEC, and treatment decisions were made empirically by attending physicians. General indications for surgery included diffuse intestinal pneumatosis, portal venous gas, progressive intestinal pneumatosis, and clinical deterioration such as abdominal wall erythema and fixed abdominal masses. Pneumoperitoneum was defined as the absolute indication for emergency laparotomy.

After 2017, we implemented a standardized protocol using a 13-point scoring system to guide surgical decision-making (Table 1). Surgical exploration was recommended for infants with three or more positive indicators on the scoring scale. The scoring system was generated by noting the presence or absence on provider the13 parameters, including clinical characteristics, radiographic and laboratory variables. A normal parameter finding was assigned a value of 0 and an abnormal finding a value of 1. Examinations were recorded at times closest to 6, 12 after the clinical diagnosis of NEC. All clinical and laboratory findings in this study were recorded at the time of initial NEC diagnosis, or surgical interventions required.

**Table 1:** Criteria for 13 score scale indicators.

Indicator	Assessment criteria	Scoring criteria
Clinical		
Palpable abdominal mass	Fixed persistent mass on serial examinations	Yes = 1
Abdominal erythema		Yes = 1
Hypotension	MAP < GA OR on any pressor	Yes = 1
Radiographic		
Pneumoperitoneum		Yes = 1
Portal venous gas		Yes = 1
Fixed loop on x-ray		Yes = 1
Severe pneumatosis	Diffuse pneumatosis or focal pneumatosis in all quadrants	Yes = 1
Laboratory		
Bacteremia	Positive blood culture within 96 h of diagnosis of NEC	Yes = 1
Hyponatremia	Sodium <130 mEq/L within last 24 h	Yes = 1
Acidosis	pH <7.25 or receiving bicarb/THAM	Yes = 1
Thrombocytopenia	Platelet count <50,000 per mm3	Yes = 1
Neutropenia	Absolute neutrophil count <2,000 per mm3	Yes = 1
Bandemia	Absolute number of immature neutrophils/total number of neutrophils >0.2	Yes = 1
Total Score		(0–13)

MAP, mean arterial pressure; THAM, trishydroxymethyl aminomethane.

Surgical approaches were selected according to intraoperative findings and surgeon preference, including intestinal resection with primary anastomosis or temporary enterostomy, and simple enterostomy. Peritoneal drainage was routinely placed after laparotomy. During surgery, all clearly necrotic intestinal segments were resected, followed by enterostomy or intestinal anastomosis. Stoma reversal was scheduled 3 months after the initial operation based on each infant's clinical condition. All enrolled infants received regular outpatient follow-up for at least 6 months after stoma closure. In this study, we compared clinical outcomes across different severity groups under the standardized postoperative management protocol.

### Data collection

We systematically extracted electronic medical records, including baseline demographic characteristics, preoperative physical examination results, radiographic images and laboratory parameters. Intraoperative information such as surgical procedures, range of intestinal resection and pathological results of resected bowel segments were also documented.

All surgical candidates were evaluated using the previously reported 13-point clinical, radiographic and laboratory scoring system for NEC surgery (Table 1) (9). Meeting all three positive criteria constitutes the minimum threshold for surgical indication. To analyze all surgically treated children and identify disparities across distinct surgical indications, drawing on our team's prior experience with multivariate regression analyses, we empirically set a cutoff score of 5 based on preliminary calculations and stratified all cases into two groups for comparative analysis. A flowchart of participant recruitment is presented in Figure 1.

**Figure 1:**
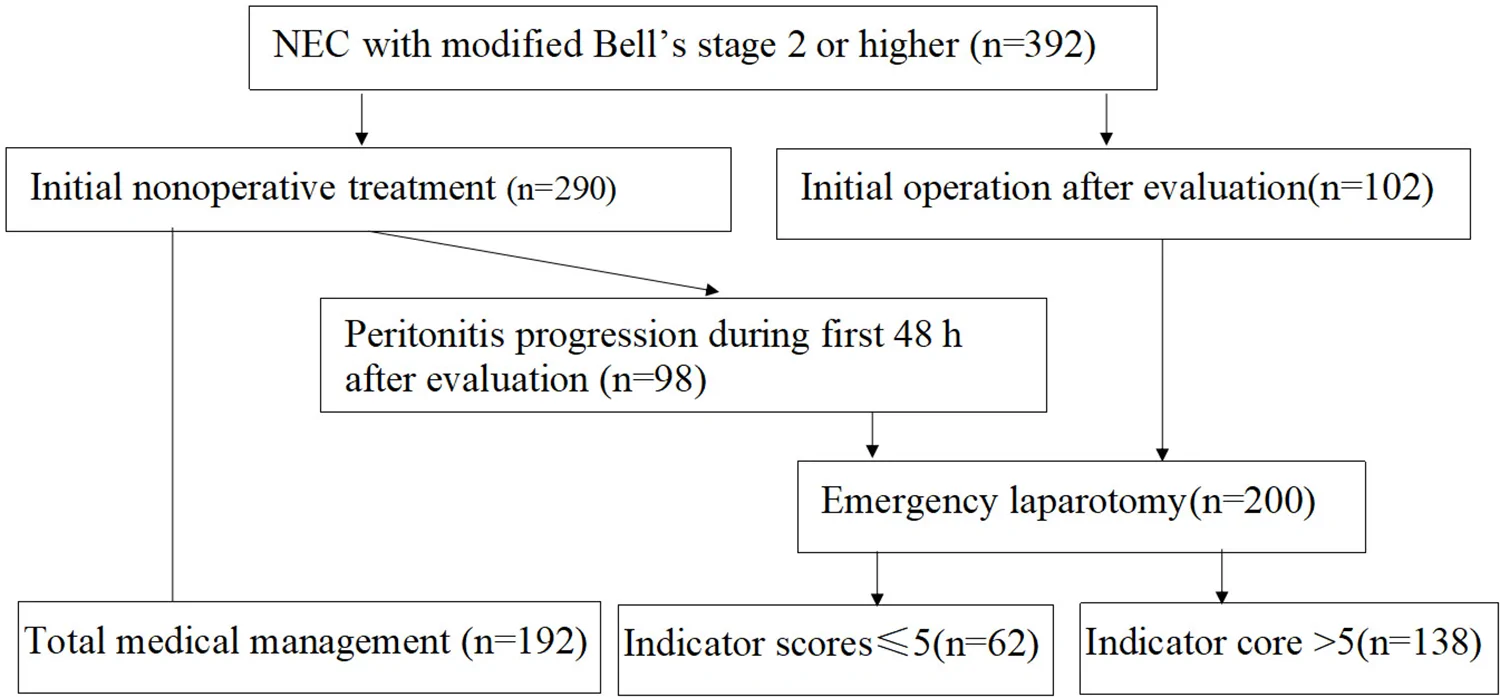
Flowchart of participant recruitment and grouping in this study.

The primary outcomes were the incidence of postoperative intestinal stricture and total bowel resection during follow-up. Intestinal stricture was diagnosed based on typical clinical manifestations (feeding intolerance, abdominal distension) and characteristic radiographic findings. The secondary outcomes included the rate of 90-day parenteral nutrition dependence, and length of NICU stay among infants surviving beyond 90 days postoperatively.

### Statistical analysis

All statistical analyses were performed using SPSS 17.0 (SPSS Inc., Chicago, IL, USA). Categorical variables (including the incidence of intestinal stricture and bowel resection) were compared using the Chi-square test or Fisher's exact test. Relative risk and odds ratio (OR) were calculated for postoperative categorical outcomes. Continuous variables with a normal distribution were analyzed using the independent samples t-test, while non-normally distributed data were compared using the Mann–Whitney U test. Univariable analysis was performed to identify the association of all predictor variables with the bowel resection. The binary multivariable unconditional logistic regression analysis was further per formed with the variables in univariable analysis (*p* < 0.2).

A two-tailed 95% confidence interval (CI) was applied for all analyses. A *P* value < 0.05 was considered statistically significant.

## Results

A total of 806 infants with NEC were screened during the study period. Among them, 439 cases met the preliminary inclusion criteria; 36 cases were excluded due to incomplete data, and another 11 were excluded because of death caused by non-intestinal complications. Ultimately, 392 infants were included in the final analysis. Ninety-eight infants underwent emergency laparotomy due to progressive deterioration after initial conservative management, including recurrent bradycardia, apnea, temperature instability and hypotension.

Overall, 192 infants received exclusive medical management, and the remaining 200 underwent surgical intervention. According to the 13-point indicator score, the surgical cohort was divided into the score ≤ 5 group (*n* = 62) and the score > 5 group (*n* = 138) (Figure 1). Baseline clinical characteristics of the three groups are summarized in Table 2. The median indicator score in the medical management group was significantly lower than that in the score > 5 group, while no significant difference was observed between the medical group and the score ≤ 5 group. Infants in the score > 5 group presented with more severe inflammatory responses, reflected by elevated white blood cell (WBC) count, C-reactive protein (CRP) and procalcitonin (PCT) levels. In addition, infants in the score > 5 group had significantly lower gestational age, birth weight and body weight at diagnosis (all *P* < 0.01).

**Table 2:** Baseline demographics for the infants among the three groups.

Characteristics	Medical Management (192)	Indicator scores ≤ 5 (62)	Indicator scores > 5 (138)
Indicator scores, median (IQR)	3 (1–6)^a^	4 (3–5)‡	5 (5–13)
Male: female	112:80	36:26	80:58
Gestational age(wk), median (IQR)	35.5 (29.2∼37.4)^a^	35.5 (30.9∼36.7)^a^	29.5 (27.9∼33.8)
Birth weight(g), median (IQR)	2.7 (2.1∼3.3)^a^	2.6 (2.0∼3.2)^a^	2.2 (1.8∼3.1)
Weight at diagnosis(g), median (IQR)	2.8 (2.3∼3.6)^a^	2.7 (2.3∼3.7)†	2.3 (2.0∼3.3)
Probiotics, *n* (%)	102 (53.1)	34 (54.8)	78 (56.5)
Vasopressor use, *n* (%)	19 (9.9%)	7 (11.3%)	19 (13.8)
Received enteral nutrition before diagnosis of NEC, *n* (%)	135 (70.3%)	45 (72.6%)	98 (71.0)
Age at diagnosis of NEC(days), median (IQR)	14 (6.5–45.5)	13 (5–35.8)	11 (5–41.6)
APGAR scores at 5 minutes, median (IQR)	10 (9∼10)	9 (9∼10)	9 (8∼10)
Cesarean delivery, *n* (%)	93 (48.4)	32 (51.6)	66 (47.8)
Maternal age(years), median (IQR)	29 (24∼39)	28 (23∼37)	28 (22∼41)
First platelet count (109/L)(onset of symptoms), mean ± SD	186.9 ± 90.3	178.5 ± 88.4	165.8 ± 93.2
Hemoglobin (g/L)(onset of symptoms), mean ± SD	13.4 ± 4.6	12.6 ± 3.7	11.3 ± 3.9
First WBC (109/L) (onset of symptoms), mean ± SD	16.6 ± 7.8	17.9 ± 8.7	18.4 ± 9.5
First PCT (onset of symptoms) (ng/mL, normal value: 0–0.5), mean ± SD	6.5 ± 3.6^a^^,^^b^	7.8 ± 4.2^a^	9.2 ± 6.3
First CRP (onset of symptoms) (mg/L, normal value: 0–10), mean ± SD	18.1 ± 8.8^a^	20.1 ± 11.6^a^	25.7 ± 13.7
Albumin (g/L, normal range, 35–50), mean ± SD	33.9 ± 6.4	31.5 ± 7.9	30.2 ± 7.5
Preoperative (Bedside) Peritoneal Drainage, *n* (%)	14 (7.3%)	11 (17.7%)	22 (15.9%)

a *p* < 0.05, Comparison with Indicator scores >5 group.

b *p* < 0.05, Comparison with Medical Management group.

No significant differences were detected across the three groups in terms of gender distribution, 5-minute APGAR score, age at NEC diagnosis, history of enteral feeding, maternal age, mode of delivery, probiotic use, vasopressor administration at disease onset, and prevalence of congenital malformations (Table 2).

### Intraoperative characteristics

Intraoperative parameters of the 200 surgical infants are shown in Table 3. The score > 5 group had a significantly higher rate of necrotic bowel resection (76.1% vs. 58.1%, *P* < 0.001) and longer median length of resected intestine (19 cm vs. 8 cm, *P* = 0.002). Resection of the ileocecal valve was also far more common in the score > 5 group (57.2% vs. 19.4%, *P* < 0.001). Infants in the score ≤ 5 group were more likely to receive simple enterostomy (30.6% vs. 15.9%, *P* = 0.023).

**Table 3:** The initial surgical features of patients who underwent laparotomy (chi-square test & Student’s t-test).

Characteristics	Indicator scores≤5 (62)	Indicator scores >5 (138)	p	Risk ratio (95% CI)
Bowel resection following ostomy, *n* (%)	36 (58.1)	105 (76.1)	<0.001	0.04 (0.02–0.09)
Bowel resection following anastomosis, *n* (%)	7 (11.3)	9 (6.5)	0.27	
Simple ostomy, *n* (%)	19 (30.6)	22 (15.9)	0.023	2.33 (1.15–4.72)
Bowel resected(cm), median (IQR)	8 (3–31)	19 (10–106)	0.002	
Resection of ileocecal valve, n%	12 (19.4%)	79 (57.2%)	<0.001	0.19 (0.09–0.38)
Operative time (mins), mean ± SD	65.9 ± 21.4	72.6 ± 27.8	0.063	
Operative blood loss (mL), median (IQR)	9 (3–31)	11 (5–45)	0.101	
Transfused patients, *n* (%)	36 (58.1)	91 (65.9)	0.027	0.52 (0.29–0.92)
Bowel involvement, *n* (%)				
Distal ileum	58 (93.5)	138 (100)	0.009	0.94 (0.88–1.00)
Distal ileum and colon	15 (24.2)	46 (33.3)	0.25	
Colon	8 (12.9)	48 (34.8)	0.001	0.28 (0.12–0.63)
Extent of disease, *n* (%)				
Multifocal NEC	8 (12.9)	46 (33.3)	0.003	0.30 (0.13–0.67)
Localized NEC	51 (82.3)	23 (16.7)	<0.001	23.18 (10.51–51.11)
Panintestinal NEC	3 (4.8)	69 (50.0)	<0.001	0.05 (0.02–0.17)

Intraoperative findings revealed multifocal or pan-intestinal lesions in most infants of the score > 5 group, whereas localized lesions predominated in the score ≤ 5 group. The distal ileum was the most frequently affected intestinal segment, followed by the ascending and descending colon. Although the score > 5 group had longer operative time and greater intraoperative blood loss, the differences did not reach statistical significance (*P* = 0.063 and *P* = 0.101, respectively). The transfusion rate was significantly higher in the score > 5 group (*P* = 0.027).

### Clinical outcomes

Postoperative outcomes are compared in Table 4. The interval between initial surgery and stoma reversal was comparable between the two surgical subgroups. The incidence of intestinal stricture during stoma reversal was significantly higher in the score > 5 group (42.0% vs. 17.7%). Accordingly, the rate of secondary total bowel resection and the average length of resected bowel were also increased in this group.

**Table 4:** Outcome characteristics in comparison between indicator scores≤5 and indicator scores >5 groups.

Characteristics	Medical Management (192)	Indicator scores ≤ 5 (62)	Indicator scores > 5 (138)
Time from primary operation to stoma reversal(days), median (IQR)	-	93 (68–179)	88 (73–196)
Intestinal stricture, *n* (%)	49 (25.5)	11 (17.7)	58 (42.0)^a^^,^^b^
Short bowel syndrome, *n* (%)	0	0	9 (6.5)
90 ds parenteral nutrition depend, *n* (%)	28 (14.6)	5 (8.1)^b^	22 (15.9)^b^
NICU length of stay (days), mean ± SD	17.2 ± 12.1	23.2 ± 13.5	26.2 ± 17.8^b^
Readmission event, *n* (%)	32 (16.7)	12 (19.4)	38 (27.5)^a^^,^^b^
Mortality, *n* (%)	0 (0)	1 (1.6)	26 (18.8)^a^^,^^b^

a *p* < 0.05, Comparison with Indicator scores ≤5 group.

b *p* < 0.05, Comparison with Medical Management group.

In the medical management group, 49 infants (25.5%) developed intestinal stricture during follow-up, mainly presenting with feeding intolerance (*n* = 37), abdominal pain (*n* = 2) and abdominal distension (*n* = 1). Most strictures were mild and only identified on imaging, while severe intestinal obstruction required secondary surgical intervention. The incidence of intestinal stricture was similar between the medical group and the score ≤ 5 group, but markedly higher in the score > 5 group.

Infants in the score > 5 group had higher rates of 90-day parenteral nutrition dependence, hospital readmission and all-cause mortality compared with the other two groups. The main causes of death in the surgical cohort were severe sepsis secondary to intestinal perforation and peritonitis (*n* = 20) and complicated short bowel syndrome (*n* = 7). No death occurred after stoma closure in any group.

To identify the potential variables for bowel resection, a comprehensive comparison of the Laboratory, Physical examination, AUS, AR features was explored between intestinal resection and no intestinal resection group. As shown in Table 2, Univariate assessment indicated that it was statistically significant for gestational age, birth weight, bacteremia, acidosis, as well as the predicted score > 5 (Table 5), which could significantly indicate bowel loss with a relatively high accuracy. The results of univariate analysis were tested by collinearity diagnosis, and the results showed that the VIF values were all less than 3, which proved that there was no significant collinearity between the data.

**Table 5:** Univariable logistic regression analysis for bowel resection.

Characteristics	Regression coefficient(*β*)	OR	95%CI	*P* value
Gestational age(wk) (Cut-off, 33.2 wk)	1.38	2.33	1.23–5.78	0.0016
Birth weight (g) (Cut-off, 2,463)	1.57	3.42	2.28–7.98	<0.001
PCT(ug/L)(Cut-off, 4.26)	2.36	4.57	4.11–11.27	0.0035
Bacteremia	3.26	4.78	2.28–13.56	<0.001
Thrombocytopenia	1.86	9.42	3.68–21.43	0.0024
Respiratory alkalosis	1.54	4.23	1.15–8.97	<0.001
Acidosis	2.68	14.51	5.23–31.54	0.0042
Peritonitis	4.83	6.64	3.54–13.87	<0.001
Abdominal erythema	2.65	6.83	1.75–23.58	0.0026
Portal venous gas	4.68	9.78	4.62–31.76	0.015
pneumoperitoneum	3.75	6.73	2.46–14.08	0.0018
Score > 5	7.87	17.45	9.86–68.46	<0.001

c:variance inflation factor.

Multivariable logistic regression was adopted further to determine the independent risk factors for bowel loss utilizing a hierarchical forward stepwise approach with the variables introduction that were determined in the univariable analysis. As shown in Table 6, five independent risk factors related to bowel loss were confirmed, including Birth weight (g) (Cut-off, 2,463) (OR, 5.14; 95% CI, 0.93–16.22; *p* = 0.014), Acidosis (OR, 3.87; 95% CI, 1.02–26.54; *p* = 0.0061), Peritonitis (OR, 11.74; 95% CI, 2.11–37.45; *p* < 0.001), and score > 5 (OR, 18.55; 95% CI, 3.78–96.32; *p* < 0.001), which were independent predictors of bowel loss.

**Table 6:** Multivariable models for the bowel resection.

Independent risk factors	Regression coefficient(β)	OR	95%CI	*P* value
Birth weight (g) (Cut-off, 2,463)	1.58	5.14	0.93–16.22	0.014
Acidosis	1.95	3.87	1.02–26.54	0.0061
Score > 5	2.96	18.55	3.78–96.32	<0.001
Peritonitis	2.12	11.74	2.11–37.45	<0.001

In this group of patients, 157 cases (78.5%) of surgical NEC involved in the small bowel resection. The intraoperative photographs were presented (Figure 2) with one infant with score ≤ 5 received early operation and only limited small bowel resection and another infant with score > 5 received delayed surgery and suffered extensive total bowel resection (Table 7).

**Figure 2:**
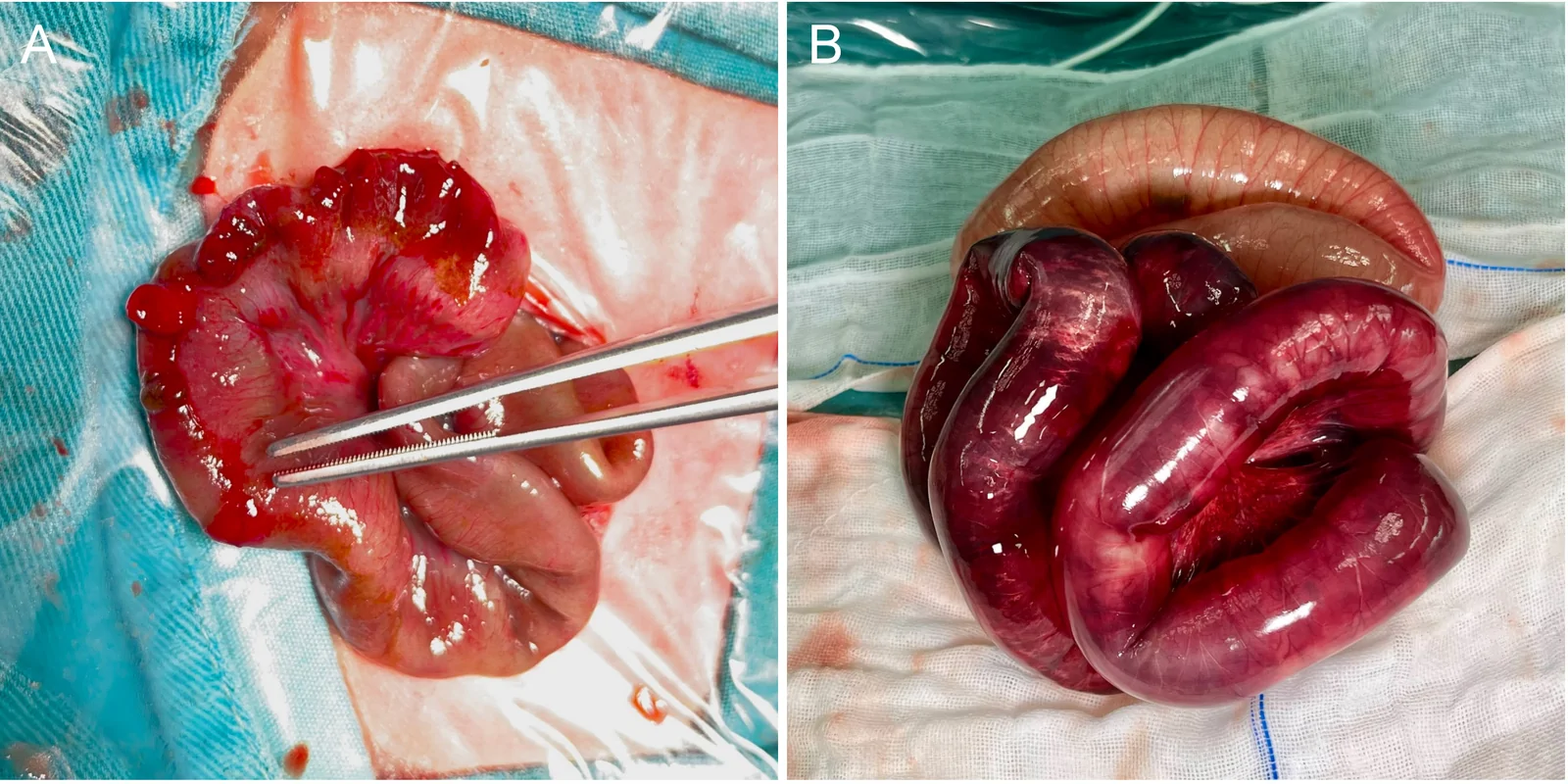
Standard intraoperative photographs showing localized bowel necrosis **(A)** vs. diffuse pan-enteric necrosis **(B)**.

**Table 7:** Clinical features of two cases with NEC.

Characteristics	Case A	Case B
Gestational age(wk)	31.5	30
Birth weight(g)	2,438.5	2,062.4
Weight at diagnosis(g)	2,518.6	2,133.9
Age at diagnosis of NEC(days)	17	22
First platelet count (10^9^/L)(onset of symptoms)	116.2	87.8
Hemoglobin (g/L)(onset of symptoms)	14.2	12.9
First WBC (10^9^/L) (onset of symptoms)	17.4	18.3
First PCT (onset of symptoms) (ng/mL, normal value: 0–0.5)	2.7	5.2
First CRP (onset of symptoms) (mg/L, normal value: 0–10)	56.5	174.1
Indicator scores	5	12

## Discussion

The present study demonstrated that early surgical intervention guided by a standardized severity scoring system can effectively reduce bowel loss, shorten NICU stay, and decrease long-term parenteral nutrition dependence in infants with severe NEC. Although infants receiving exclusive conservative management did not undergo initial bowel resection, their overall incidence of intestinal stricture was higher than that of infants who received timely surgery with low indicator scores. These findings confirm that appropriate early surgical intervention helps minimize total bowel loss and improves postoperative recovery in infants with NEC.

Approximately 20% to 40% of infants with NEC require surgical treatment (10, 11). Despite accumulated clinical experience, there remains no global consensus on the optimal timing of surgery for NEC (12, 13). Timely surgical exploration is life-saving for critically ill infants with advanced NEC and contributes to improved overall prognosis (14–16). However, overly aggressive early surgery may also lead to adverse complications (17). Therefore, accurate identification of surgical timing is critical, and clinical decisions currently rely heavily on individual surgeon experience, which directly affects patient outcomes. In this retrospective analysis, we evaluated the correlation between surgical timing and two key endpoints: intestinal stricture and total bowel loss.

During the observation period, 98 infants initially managed conservatively progressed to severe NEC and eventually required emergency surgery. Close monitoring of clinical changes and prompt surgical intervention contributed to better outcomes in infants with low indicator scores, highlighting the advantages of standardized early surgical strategies.

Preserving functional intestinal length is another core goal of NEC surgery, as extensive intestinal resection caused by progressive ischemia and perforation leads to poor long-term outcomes (18, 19). Our data verified that early surgical intervention is associated with reduced bowel loss. The higher baseline disease severity in the high-score group may partially explain increased bowel loss, and surgical timing is only one of the independent influencing factors rather than the sole determinant. The rate of total bowel resection was 20.3% in the medical management group, 21.0% in the score ≤ 5 surgical group, and 52.2% in the score > 5 group with delayed surgery.

Post-NEC intestinal stricture results from intestinal ischemia and tissue fibrosis, and is a major cause of secondary bowel loss (20, 21). Our results showed a positive correlation between delayed intervention/conservative management and stricture formation. The incidence of intestinal stricture was 25.5% in the medical group and 17.7% in the early surgery group. Even mild strictures detected on imaging may progress to symptomatic obstruction and require reoperation. Given the high risk of secondary bowel loss associated with strictures, timely surgical consultation is essential to optimize long-term intestinal outcomes. At present, evidence linking surgical delay to increased bowel loss in NEC remains insufficient (22, 23). Further research should focus on the long-term benefits of standardized early surgical protocols for bowel preservation. Previous studies have also reported that the incidence of colonic stricture is not affected by the type of surgical procedure performed (24, 25).

### Study limitations

Several limitations of this study should be acknowledged. First, this is a retrospective multicenter observational study; variations in clinical practice, surgical proficiency and perioperative care across participating centers may introduce unmeasured confounding factors. Second, the implementation of the 13-point scoring protocol after 2017 changed surgical decision-making criteria during the study period, leading to temporal bias. Third, treatment allocation was non-randomized: surgical intervention was determined by clinical indications and surgeon judgment rather than random grouping, resulting in inherent selection bias. Fourth, significant baseline differences in disease severity, gestational age and birth weight existed across groups, which may interfere with the association between surgical timing and clinical outcomes. Fifth, surgical thresholds varied between individual surgeons due to the lack of fully automated standardized decision systems. Finally, residual confounding from uncollected clinical variables cannot be completely excluded despite statistical adjustment. For the above reasons, our findings are hypothesis-generating, and further prospective controlled studies are required for external validation. This retrospective study cannot establish definitive causality, and baseline prematurity, low birth weight, and elevated inflammatory markers are inherent confounding factors that may affect bowel injury severity and resection extent. We acknowledge that excluding infants who died within 30 days due to non-intestinal complications may introduce survival bias by removing the most critically ill patients.

## Conclusions

In conclusion, early surgical intervention guided by the 13-point scoring system helps reduce bowel loss in infants with severe NEC. Although causal relationships cannot be established due to baseline imbalance and non-random treatment assignment, our results suggest that surgical intervention is recommended when the indicator score exceeds 5 to optimize long-term intestinal outcomes. Large-scale prospective studies are warranted to validate these conclusions.

## Data Availability

The raw data supporting the conclusions of this article will be made available by the authors, without undue reservation.

## References

[1] Perrone S Cremonini I Marinelli F Monaco S Nicoletti L Giordano M. New strategies for necrotizing enterocolitis diagnosis and prevention in newborns. Curr Pediatr Rev. (2021) 17:191–200. 10.2174/1573396317666210426102610 33902422

[2] Nair J Maheshwari A. Epigenetics in necrotizing enterocolitis. Curr Pediatr Rev. (2021) 17:172–84. 10.2174/1573396317666210421110608 33882811

[3] El Manouni El Hassani S Niemarkt HJ Derikx JPM Berkhout DJC Ballón AE De Graaf M. Predictive factors for surgical treatment in preterm neonates with necrotizing enterocolitis: a multicenter case-control study. Eur J Pediatr. (2021) 180(2):617–25. 10.1007/s00431-020-03892-1 33269424 PMC7813726

[4] Robinson JR Rellinger EJ Hatch LD Weitkamp J-H Speck KE Danko M. Surgical necrotizing enterocolitis. Semin Perinatol. (2017) 41(1):70–9. 10.1053/j.semperi.2016.09.020 27836422 PMC5777619

[5] Tepas JJ 3rd Sharma R Leaphart CL Celso BG Pieper P Esquivia-Lee V Timing of surgical intervention in necrotizing enterocolitis can be determined by trajectory of metabolic derangement. J Pediatr Surg. 2010;45(2):310–3. 10.1016/j.jpedsurg.2009.10.069 20152342

[6] Yu M Liu G Feng Z Huang L. Combination of plasma white blood cell count, platelet count and C-reactive protein level for identifying surgical necrotizing enterocolitis in preterm infants without pneumoperitoneum. Pediatr Surg Int. (2018) 34(9):945–50. 10.1007/s00383-018-4305-6 30027466

[7] Petty JK Ziegler MM. Operative strategies for necrotizing enterocolitis: the prevention and treatment of short-bowel syndrome. Semin Pediatr Surg. (2005) 14(3):191–8. 10.1053/j.sempedsurg.2005.05.009 16084407

[8] Berman L Moss RL. Necrotizing enterocolitis: an update. Semin Fetal Neonatal Med. (2011) 16(3):145–50. 10.1016/j.siny.2011.02.002 21514258

[9] Munaco AJ Veenstra MA Brownie E Danielson LA Nagappala KB Klein MD. Timing of optimal surgical intervention for neonates with necrotizing enterocolitis. Am Surg. (2015) 81(5):438–43. 10.1177/000313481508100521 25975324

[10] Wales PW De Silva N Kim JH Lecce L Sandhu A Moore AM. Neonatal short bowel syndrome: a cohort study. J Pediatr Surg. (2005) 40(5):755–62. 10.1016/j.jpedsurg.2005.01.037 15937809

[11] Bull KE Gainey AB Cox CL Burch AK Durkin M Daniels R. Evaluation of time to resolution of medical necrotizing enterocolitis using severity-guided management in a neonatal intensive care unit. J Pediatr Pharmacol Ther. (2021) 26(2):179–86. 10.5863/1551-6776-26.2.179 33603582 PMC7887889

[12] D Bernardo G Sordino D D Chiara C Riccitelli M Esposito F Giordano M. Management of NEC: surgical treatment and role of traditional x-ray versus ultrasound imaging, experience of a single centre. Curr Pediatr Rev. (2019) 15(2):125–30. 10.2174/1573396314666181102122626 30387397

[13] Geng Q Wang Y Li L Guo C. Early postoperative outcomes of surgery for intestinal perforation in NEC based on intestinal location of disease. Medicine (Baltimore). (2018) 97(39):e12234. 10.1097/MD.0000000000012234 30278493 PMC6181543

[14] Wang H Wang Y Deng C Li L Guo C. Prediction of intestinal failure from necrotizing enterocolitis following surgery: a multicenter retrospective review. Medicine (Baltimore). (2019 May) 98(19):e15568. 10.1097/MD.0000000000015568 PMC6531265 31083227

[15] Sapin E Carricaburu E Boissieu D Goutail-Flaud D Benammar MF Helardot S. Conservative intestinal surgery to avoid short-bowel syndrome in multiple intestinal atresias and necrotizing enterocolitis: 6 cases treated by multiple anastomoses and santulli-type enterostomy. Eur J Pediatr Surg. (1999) 9(1):24–8. 10.1055/s-2008-1072207 10207699

[16] Romão RL Ozgediz D De Silva N Chiu P Langer J Wales PW. Preserving bowel length with a transluminal stent in neonates with multiple intestinal anastomoses: a case series and review of the literature. J Pediatr Surg. (2011) 46(7):1368–72. 10.1016/j.jpedsurg.2011.01.011 21763836

[17] GaŁĄzka P Chrzanowska M StyczyŃski J. Clinical Spectrum and outcomes of neonatal necrotizing enterocolitis. In Vivo. (2021) 35(1):585–91. 10.21873/invivo.12295 PMC7880741 33402513

[18] Sparks EA Khan FA Fisher JG Fullerton BS Hall A Raphael BP. Necrotizing enterocolitis is associated with earlier achievement of enteral autonomy in children with short bowel syndrome. J Pediatr Surg. (2016) 51(1):92–5. 10.1016/j.jpedsurg.2015.10.023 26700691 PMC4878438

[19] Mangalat N. Pediatric intestinal failure: a review of the scope of disease and a regional model of a multidisciplinary care team. Mo Med. (2019) 116(2):129–33. 31040499 PMC6461325

[20] Patel EU Wilson DA Brennan EA Lesher AP Ryan RM. Earlier re-initiation of enteral feeding after necrotizing enterocolitis decreases recurrence or stricture: a systematic review and meta-analysis. J Perinatol. (2020 Nov) 40(11):1679–87. 10.1038/s41372-020-0722-1 PMC7368613 32683411

[21] Die X Cui M Feng W Hou J Chen P Liu W. Applications of indocyanine greenenhanced fluorescence in the laparoscopic treatment of colonic stricture after necrotizing enterocolitis. BMC Pediatr. (2023) 23(1):635. 10.1186/s12887-023-04458-4 38102599 PMC10724931

[22] Abdullah F Zhang Y Camp M Mukherjee D Gabre-Kidan A Colombani PM. Necrotizing enterocolitis in 20,822 infants: analysis of medical and surgical treatments. Clin Pediatr (Phila). (2010) 49(2):166–71. 10.1177/0009922809349161 20080523

[23] Liu W Wang Y Zhu J Zhang C Liu G Wang X. Clinical features and management of post-necrotizing enterocolitis strictures in infants: a multicentre retrospective study. Medicine (Baltimore). (2020) 99(19):e20209. 10.1097/MD.0000000000020209 32384517 PMC7220416

[24] Zhang H Chen J Wang Y Deng C Li L Guo C. Predictive factors and clinical practice profile for strictures post-necrotising enterocolitis. Medicine (Baltimore). (2017) 96(10):e6273. 10.1097/MD.0000000000006273 28272242 PMC5348190

[25] Heida FH Loos MH Stolwijk L T Kiefte BJ Van Den Ende SJ Onland W. Risk factors associated with postnecrotizing enterocolitis strictures in infants. J Pediatr Surg. (2016) 51(7):1126–30. 10.1016/j.jpedsurg.2015.09.015 26472655

